# Supplementary material for: The Contribution of Thai Fisheries to Sustainable Seafood Consumption: National Trends and Future Projections
Source: Foods. 2021 Apr 17;10(4):880. doi: 10.3390/foods10040880 (PMC8073281; doi:10.3390/foods10040880)
Supplement: Supplementary file 1 [file foods-10-00880-s001.zip › foods-1156383-supplementary.pdf]

Supplementary Materials

The contribution of Thai fisheries to sustainable seafood consumption: national trends and future projections

Tiptiwa Sampantamit[1, 2,*], Long Ho[1*], Carl Lachat[3], Giles Hanley-Cook[3], and Peter Goethals[1]

[1] Department of Animal Sciences and Aquatic Ecology, Faculty of Bioscience Engineering, Ghent University, 9000 Ghent, Belgium; Long.TuanHo@UGent.be (L.H.); Peter.Goethals@UGent.be (P.G.)
[2] Department of Biological and Environmental Sciences, Faculty of Science, Thaksin University, 93110 Patthalung, Thailand
[3] Department of Food Technology, Safety and Health, Ghent University, 9000 Ghent, Belgium; Carl.Lachat@UGent.be (C.L.); Giles.HanleyCook@UGent.be (G.H-C.)

*Correspondence: Tiptiwa@tsu.ac.th; Long.tuanho@UGent.be

Table S1. Nutrient composition of fish and shellfish products

No.	English name	Scientific name	Family	% Edible portion of whole fish or shellfish	Nutrition content per 100g edible raw portion				Source of nutrient composition of fish and shellfish products
					Protein (g)	Ca (mg)	Fe (mg)	Zn (mg)	
	Fish								
1	Short mackerel	*Rastrelliger brachysoma*	Scombridae	57	21.4	62	1.4	0.6	Institute of Nutrition, Mahidol University (2014)
2	Indian mackerel	*Rastrelliger kanagurta*	Scombridae	57	21.5	48	1.8	0.6	Institute of Nutrition, Mahidol University (2014)
3	Narrow-barred Spanish mackerel	*Scomberomorus commerson*	Scombridae	67	19.4	17	0.8	0.4	Institute of Nutrition, Mahidol University (2014)
4	Wolf-herrings	*Chirocentrus* spp.	Chirocentridae	60	21.8	23	0.5	0.6	Siong et al. (1987); Tacon and Metian (2013)
5	Longtail tuna	*Thunnus tonggol*	Scombridae	58	24.4	4	0.8	0.4	USDA (2019)
6	Eastern little tuna	*Euthynnus affinis*	Scombridae	58	22	29	1.3	0.8	USDA (2019)
7	Round scads	*Decapterus* spp.	Carangidae	49	20.4	61	1.2	0.3	Food and Nutrition Research Institute, Department of Science and Technology (2019)

No.	English name	Scientific name	Family	% Edible portion of whole fish or shellfish	Nutrition content per 100g edible raw portion				Source of nutrient composition of fish and shellfish products
					Protein (g)	Ca (mg)	Fe (mg)	Zn (mg)	
8	Hardtail scad	*Megalaspis cordyla*	Carangidae	42	21.3	58	2.3	0.3	Institute of Nutrition, Mahidol University (2014)
9	Trevallies	*Selaroides leptolepis*	Carangidae	48	21.4	54	0.9	0.3	Siong et al. (1987); Institute of Nutrition, Mahidol University (2014)
10	Big-eye scad	*Caranx sexfasciatus*	Carangidae	44	21.5	80	0.8	0.3	Siong et al. (1987); Institute of Nutrition, Mahidol University (2014)
11	Black banded kingfish	*Seriolina nigrofasciata*	Carangidae	48	18.6	42	0.6	0.3	Institute of Nutrition, Mahidol University (2014)
12	Threadfin	*Eleutheronema tetradactylum*	Polynemidae	57	20.2	32	0.5	0.6	Institute of Nutrition, Mahidol University (2014); Tacon and Metian (2013)
13	Sardines	*Sardinella* spp.	Clupeidae	65	17.5	80	3	0.9	National Institute of Nutrition (2007)
14	Anchovies	*Stolephorus* spp.	Engraulidae	100	18.5	168	1	0.7	Institute of Nutrition, Mahidol University (2014)
15	Mullet	*Liza* spp.	Mugilidae	50	24.7	94	4.3	0.5	Institute of Nutrition, Mahidol University (2014)
16	Black pomfret	*Parastromateus niger*	Carangidae	49	19.8	43	0.6	0.3	Institute of Nutrition, Mahidol University (2014)
17	Silver pomfret	*Pampus argenteus*	Stromateidae	58	19.4	15	0.6	0.3	Institute of Nutrition, Mahidol University (2014)
18	Barrcudas	*Sphyraena* spp.	Sphyraenidae	58	17.6	11.4	0.5	0.4	Nutrition division (2001); Longvah et al. (2017)
19	Tunas	*Thunnus* spp.	Scombridae	58	24.4	4	0.8	0.4	USDA (2019)
20	Croaker	*Johnius* spp.	Sciaenidae	50	18.6	32	0.4	0.7	Institute of Nutrition, Mahidol University (2014)
21	Threadfin breams	*Nemipterus hexodon*	Nemipteridae	50	18.1	46	0.5	0.4	Ministry of Education Culture Sports Science and Technology (2005)
22	Monocle breams	*Scolopsis* spp.	Nemipteridae	50	18.1	46	0.5	0.4	Ministry of Education Culture Sports Science and Technology (2005)

No.	English name	Scientific name	Family	% Edible portion of whole fish or shellfish	Nutrition content per 100g edible raw portion				Source of nutrient composition of fish and shellfish products
					**Protein (g)**	**Ca (mg)**	**Fe (mg)**	**Zn (mg)**	
23	Lizard fish	*Saurida* spp.	Synodontidae	55	20.1	80	0.3	0.4	Ministry of Education Culture Sports Science and Technology (2005)
24	Hairtail	*Trichiurus* spp.	Trichiuridae	59	18.3	36	0.2	0.4	National Institute of Nutrition (2007); Longvah et al. (2017)
25	Red snappers	*Lutjanus argentimaculatus*	Latjanidae	50	16.9	31	0.6	0.1	Institute of Nutrition, Mahidol University (2014)
26	Sea bass	*Lates calcarifer*	Latidae	55	20.5	26	0.4	0.6	Institute of Nutrition, Mahidol University (2014); Tacon and Metian (2013)
27	Big-eyes	*Priacanthus* spp.	Priacanthidae	72	21.5	17.4	0.6	0.4	Longvah et al. (2017)
28	Sand whitings	*Sillago sihama*	Sillaginidae	51	18.5	27	0.1	0.4	Ministry of Education Culture Sports Science and Technology (2005)
29	Catfish eel	*Plotosus* spp.	Plotosidae	60	17.5	14	0.8	0.6	Siong et al. (1987); Tacon and Metian (2013)
30	Sea catfish	*Arius* spp.	Ariidae	45	21.6	14.7	0.5	0.8	Longvah et al. (2017)
31	Rays	Rays mixed species	Mixed groups	40	24.0	9.2	0.7	0.4	Longvah et al. (2017)
32	Sharks	Shark mixed species	Mixed groups	35	21.0	34	0.8	0.4	USDA (2019)
33	Flatfish	*Paraplagusia* spp.	Cynoglossidae	55	19.2	36	0.3	0.5	Ministry of Education Culture Sports Science and Technology (2005)
34	Indian halibut	*Psettodes erumei*	Psettodidae	49	20.6	43	0.4	0.3	Nutrition division (2001); Longvah et al. (2017); Siong et al. (1987)
35	Conger eel	*Muraenesox* spp. *Congresox* spp.	Muraenesocidae	60	22.3	79	0.2	0.6	Ministry of Education Culture Sports Science and Technology (2005)
36	Groupers	*Epinephelus coioides*	Serranidae	44	18.9	54	0.4	0.2	Institute of Nutrition, Mahidol University (2014)
37	Other food fish	Fish mixed species	Mixed group	39	17	16	0.3	0.6	Fellows and Hampton (1992); Tacon and Metian (2013)
38	Trash fish	Fish mixed species	Mixed group	39	17	16	0.3	0.6	Fellows and Hampton (1992); Tacon and Metian (2013)

No.	English name	Scientific name	Family	% Edible portion of whole fish or shellfish	Nutrition content per 100g edible raw portion				Source of nutrient composition of fish and shellfish products
					Protein (g)	Ca (mg)	Fe (mg)	Zn (mg)	
39	Common silver carp	*Barbonymus gonionotus*	Cyprinidae	50	21.1	32	0.6	0.1	Institute of Nutrition, Mahidol University (2014)
40	Snakeskin gourami	*Trichopodus pectoralis*	Osphronemidae	48	17.6	59	1.8	0.6	Institute of Nutrition, Mahidol University (2014); USDA (2019)
41	Walking catfish	*Clarias* spp.	Clariidae	70	17.8	20	0.8	0.2	Institute of Nutrition, Mahidol University (2014)
42	Striped snake head	*Channa striata*	Channidae	46	20.5	31	5.8	0.6	Nutrition division (2001); Tacon and Metian (2013)
43	Fish mixed group	Fish mixed group	Fish mixed group	39	17	16	0.3	0.6	Fellows and Hampton (1992); Tacon and Metian (2013)
44	Nile tilapia	*Oreochromis niloticus*	Cichlidae	30	18.3	15	0.8	0.4	FAO (2016)
45	Common carp	*Cyprinus* spp.	Cyprinidae	50	17	26	1.2	1.5	Institute of Nutrition, Mahidol University (2014)
46	Chinese major carps	Fish mixed species	Cyprinidae	50	21.1	32	0.6	0.1	Institute of Nutrition, Mahidol University (2014)
47	Striped catfish	*Pangasianodon hypophthalmus*	Pangasiidae	50	15.0	24	1.4	0.1	Institute of Nutrition, Mahidol University (2014)
48	Java tilapia	*Oreochromis mossambicus*	Cichlidae	30	18.3	15	0.8	0.4	FAO (2016)
49	Sand Goby	*Oxyeleotris marmorata*	Eleotridae	44	17.1	29	0.8	0.6	Institute for Medical Research (1997); Tacon and Metian (2013)
50	Giant qourami	*Osphronemus goramy*	Osphroneminae	48	17.6	59	1.8	0.6	Institute of Nutrition, Mahidol University (2014); Tacon and Metian (2013)
51	Swamp eel	*Monopterus albus*	Synbranchidae	52	19.7	33	1.7	0.8	Institute of Nutrition, Mahidol University (2014)
52	Roho labeo	*Labeo rohita*	Cyprinidae	50	16.9	19	1.2	0.6	Institute for Medical Research (1997); Tacon and Metian (2013)

No.	English name	Scientific name	Family	% Edible portion of whole fish or shellfish	Nutrition content per 100g edible raw portion				Source of nutrient composition of fish and shellfish products
					Protein (g)	Ca (mg)	Fe (mg)	Zn (mg)	
53	Grey featherback	*Notopterus* spp.	Notopteridae	57	19.6	77	0.3	0.6	Institute of Nutrition, Mahidol University (2014); Tacon and Metian (2013)
54	Spot pangasius	*Pangasius larnaudii*	Pangasiidae	50	16.6	11	0.5	0.6	Institute for Medical Research (1997); Tacon and Metian (2013)
55	Moonlight gourami	*Trichopodus* spp.	Osphronemidae	48	17.6	59	1.8	0.6	Institute of Nutrition, Mahidol University (2014); Tacon and Metian (2013)
56	Common climbing perch	*Anabas testudineus*	Anabantidae	44	17.9	110	1.2	0.6	Institute of Nutrition, Mahidol University (2014); Tacon and Metian (2013)
57	Giant Snakehead	*Channa micropeltes*	Channidae	46	20.5	31	5.8	0.6	Nutrition division (2001); Tacon and Metian (2013)
58	Mrigal carp	*Cirrhinus mrigala*	Cyprinidae	50	21.1	32	0.6	0.1	Institute of Nutrition, Mahidol University (2014)
	Shrimps								
1	Banana shrimp	*Penaeus merguiensis*	Penaeidae	62	19	64	1.4	1.4	Food and Nutrition Research Institute, Department of Science and Technology (2019) and USDA (2019)
2	Jumbo tiger prawn	*Penaeus monodon*	Penaeidae	63	20	65	2.2	1.4	Food and Nutrition Research Institute, Department of Science and Technology (2019)
3	White shrimp	*Litopenaeus vannamei*	Penaeidae	80	19.6	68	1.4	1.2	Ministry of Education Culture Sports Science and Technology (2005)
4	Tiger shrimp	*Penaeus monodon*	Penaeidae	63	20	65	2.2	1.4	Food and Nutrition Research Institute, Department of Science and Technology (2019)
5	king prawn	*Penaeus latisulcatus*	Penaeidae	57	23.9	45	1.4	1.5	FAO (2016)

No.	English name	Scientific name	Family	% Edible portion of whole fish or shellfish	Nutrition content per 100g edible raw portion				Source of nutrient composition of fish and shellfish products
					Protein (g)	Ca (mg)	Fe (mg)	Zn (mg)	
6	School prawn	*Metapenaeus* spp.	Penaeidae	50	18.7	56	1	1	Ministry of Education Culture Sports Science and Technology (2005)
7	Other shrimp	Shrimp mixed species	Penaeidae	57	20.1	64	0.5	1.3	Tacon and Metian (2013)
8	Aetes	*Acetes* spp.	Sargestidae	100	12	64	3.4	1.3	Ministry of Education Culture Sports Science and Technology (2005)
9	Flathead lobster	*Thenus orientalis*	Scyllarodae	30	20.6	49	1.2	0.4	USDA (2019)
10	Mantis shrimp	*Harpiosquilla raphidea, Miyakea nepa, Oratosquilla nepa*	Squillidae	30	20.6	49	1.2	0.4	USDA (2019)
11	Macrobracium	*Macrobrachium rosenbergii*	Palaemonidae	54	17.9	17	1	1.3	Institute of Nutrition, Mahidol University (2014)
12	Shrimp mixed group	Shrimp mixed group	Shrimp mixed group	57	13.6	54	1.2	3	Tacon and Metian (2013)
	Crabs								
1	Swimming crabs	*Portunus pelagicus*	Potunidae	35	16.2	127	1.7	1.7	Tacon and Metian (2013); Nutrition division (2001)
2	Mangrove crabs	*Scylla serrate*	Potunidae	42	17.9	183	2.6	3	Institute of Nutrition, Mahidol University (2014); Tacon and Metian (2013)
3	Other crabs	Crab mixed species	Mixed groups	20	17.2	153	1.2	3	FAO (2016)
	Molluscs								
1	Blood cockle	*Anadara granosa*	Arcidae	25	11.7	181	10.5	2.4	USDA (2019)
2	Green mussel	*Perna viridis*	Mytilidae	56	9.3	25	10.3	1	USDA (2019)
3	Oyster	*Saccostrea* spp.	Ostreidae	12	5.9	147	5.9	7.1	Food and Nutrition Research Institute, Department of Science and Technology (2019)
4	Horse mussel	*Musculus senhousia*	Mytilidae	56	9.3	25	10.3	1	USDA (2019)

No.	English name	Scientific name	Family	% Edible portion of whole fish or shellfish	Nutrition content per 100g edible raw portion				Source of nutrient composition of fish and shellfish products
					**Protein (g)**	**Ca (mg)**	**Fe (mg)**	**Zn (mg)**	
5	Short necked clam	*Paphia* spp.	Veneridae	27	9.2	183	6.6	1.2	FAO (2016)
6	Scallop	*Amusium* spp.	Pectinidae	13	22.3	14	0.6	0.9	USDA (2019)
7	Other shellfishes	Mussel mixed species	Mussel mixed Family	11	14.7	39	1.6	0.5	USDA (2019)
	Squids								
1	Squid	*Loligo* spp.	Loliginidae	96	15.6	32	0.7	1.5	USDA (2019)
2	Cuttlefish	*Sepia pharaonis*	Sepiidae	65	15.9	16	1.1	1.5	Institute of Nutrition, Mahidol University (2014)
3	Bigfin reef squid	*Sepioteuthis lessoniana*	Loliginidae	96	15.6	32	0.7	1.5	USDA (2019)
4	Octopus	*Octopus* spp.	Octopodidae	94	14.7	39	1.6	0.5	USDA (2019)

Table S2. Average seafood intake in 187 countries. Source: GDD (2019)

No.	List of countries	Average seafood intake (g/capita/day)
1	Guatemala	5.4
2	Zimbabwe	5.8
3	Honduras	6
4	Nicaragua	6.3
5	El Salvador	7.2
6	Lesotho	7.4
7	Botswana	7.6
8	Uzbekistan	7.7
9	Occupied Palestinian Territory	7.9
10	Timor-Leste	7.9
11	Tajikistan	8
12	Colombia	8.1
13	Lebanon	8.1
14	Costa Rica	8.1
15	Mongolia	8.4
16	Swaziland	9.2
17	Namibia	9.3
18	South Africa	9.4
19	Mexico	9.4
20	Syrian Arab Republic	9.7
21	Albania	10.2
22	Armenia	10.3
23	Kyrgyzstan	10.4
24	Bolivia	10.5
25	Azerbaijan	10.6
26	Nepal	10.8
27	Bosnia and Herzegovina	10.9
28	Eritrea	11.1
29	Panama	11.7
30	Pakistan	11.7
31	Macedonia (Former Yugoslav Republic of)	11.8
32	Venezuela (Bolivarian Republic of)	11.8
33	Hungary	12.1
34	Netherlands	12.2
35	Rwanda	12.4
36	Serbia	12.7
37	Romania	12.7
38	Bulgaria	12.8
39	Djibouti	12.9
40	Slovenia	13
41	Slovakia	13.1
42	Kazakhstan	13.2
43	Sudan	13.2
44	Afghanistan	13.3
45	Poland	13.5
46	Turkmenistan	13.7
47	Bhutan	13.9
48	Georgia	14.7
49	Argentina	14.9
50	India	15
51	Moldova	15.2
52	Jordan	15.3
53	Montenegro	15.4
54	Iraq	15.5
55	Guinea-Bissau	15.6
56	Czech Republic	15.6
57	Oman	15.7
58	Germany	15.9

No.	List of countries	Average seafood intake (g/capita/day)
59	Turkey	16
60	Belarus	16.2
61	Niger	16.2
62	Qatar	16.3
63	Uruguay	16.3
64	Ecuador	16.3
65	Mozambique	16.4
66	Bahrain	16.4
67	Canada	16.6
68	Haiti	16.7
69	Burundi	16.8
70	Croatia	16.9
71	Saudi Arabia	16.9
72	Libyan Arab Jamahiriya	17.1
73	United States of America	17.4
74	Yemen	17.7
75	Morocco	17.8
76	Burkina Faso	17.9
77	Kuwait	18.6
78	Central African Republic	19.2
79	Tunisia	19.3
80	Lithuania	19.4
81	Iran (Islamic Republic of)	19.8
82	Algeria	19.8
83	Ireland	20
84	Austria	20.3
85	Kenya	20.5
86	Ukraine	20.5
87	Egypt	20.5
88	Somalia	21
89	Estonia	21.1
90	Bangladesh	21.2
91	Dominican Republic	21.4
92	United Arab Emirates	21.4
93	Ethiopia	21.5
94	Democratic Republic of the Congo	22.4
95	Malawi	22.8
96	Australia	23
97	Cuba	23.2
98	Cyprus	23.9
99	Belgium	24
100	Mauritius	24.1
101	Israel	24.1
102	Belize	24.1
103	Switzerland	24.4
104	Latvia	24.5
105	Madagascar	24.5
106	New Zealand	24.8
107	Lao People's Democratic Republic	25.1
108	Zambia	25.1
109	Trinidad and Tobago	25.9
110	Nigeria	25.9
111	Greece	26
112	Liberia	26.3
113	Russian Federation	26.4
114	Equatorial Guinea	26.4
115	United Republic of Tanzania	26.7
116	Luxembourg	27.3
117	Viet Nam	27.3

No.	List of countries	Average seafood intake (g/capita/day)
118	Chad	27.7
119	Indonesia	27.9
120	Mali	28.3
121	Angola	28.5
122	Saint Vincent and the Grenadines	28.5
123	Myanmar	28.5
124	Andorra	28.7
125	Cambodia	28.7
126	United Kingdom	28.9
127	Uganda	29.2
128	Sweden	29.8
129	Taiwan	29.9
130	Benin	30.1
131	Sri Lanka	30.1
132	Suriname	30.3
133	Democratic People's Republic of Korea	30.3
134	Guinea	30.6
135	France	31
136	China	31.3
137	Peru	31.5
138	Italy	31.6
139	Malta	31.6
140	Thailand	32.1
141	Finland	32.1
142	Togo	33.2
143	Bahamas	33.8
144	Chile	33.9
145	Philippines	33.9
146	Dominica	33.9
147	Congo	34
148	Mauritania	35
149	Jamaica	35
150	Brazil	35.4
151	Cameroon	35.7
152	Malaysia	36
153	Paraguay	36.1
154	Cape Verde	36.7
155	Gabon	36.7
156	Cote d'Ivoire	37.2
157	Singapore	37.7
158	Fiji	38.1
159	Barbados	38.7
160	Saint Lucia	39
161	Papua New Guinea	39.3
162	Seychelles	39.7
163	Grenada	40.7
164	Sierra Leone	40.8
165	Comoros	40.9
166	Guyana	41.8
167	Vanuatu	42.2
168	Gambia	42.3
169	Micronesia (Federated States of)	42.7
170	Antigua and Barbuda	42.9
171	Marshall Islands	43.6
172	Sao Tome and Principe	44.3
173	Tonga	45.6
174	Senegal	45.9
175	Denmark	47.6
176	Solomon Islands	47.9

No.	List of countries	Average seafood intake (g/capita/day)
177	Ghana	47.9
178	Kiribati	48.6
179	Samoa	50
180	Norway	50.5
181	Iceland	50.5
182	Brunei Darussalam	51.1
183	Spain	54.9
184	Republic of Korea	55.5
185	Portugal	56
186	Maldives	61.8
187	Japan	74.9

Table S3. Total annual yields of capture fisheries and aquaculture production, amount of fish used for fish meal, and quantity of export and import of fishery products from 1995 to 2015 (kg/year). Source: DoF (1998-2017)

Year	Capture fisheries production yields (kg/year)	Aquaculture production yields (kg/year)	Quantity used in fish meal production (kg/year)	Exported fishery products (kg/year)	Imported fishery products (kg/year)
1995	3,019,147,000	553,608,000	1,796,673,000	1,191,362,000	700,437,000
1996	2,994,525,000	554,704,000	1,697,918,000	1,146,239,000	635,609,000
1997	2,884,492,000	499,912,000	1,515,757,000	1,179,903,000	644,689,000
1998	2,911,268,000	594,595,000	1,323,887,000	1,286,981,000	716,382,000
1999	2,932,107,000	693,778,000	1,202,283,000	1,389,400,000	840,804,000
2000	2,975,165,000	738,084,000	1,147,091,000	1,347,690,000	741,067,000
2001	2,834,202,000	814,245,000	1,437,731,000	1,391,912,000	869,367,000
2002	2,842,411,000	954,603,000	1,507,644,000	1,429,230,000	968,695,000
2003	2,849,623,000	1,064,403,000	1,529,028,000	1,636,426,000	1,074,680,000
2004	2,839,669,000	1,259,980,000	1,555,950,000	1,636,987,000	1,232,977,000
2005	2,814,365,000	1,304,211,000	1,554,541,000	1,732,434,000	1,436,978,000
2006	2,698,803,000	1,354,277,000	1,405,305,000	1,877,047,000	1,468,074,000
2007	2,304,951,000	1,370,426,000	1,312,266,000	1,829,109,000	1,394,669,000
2008	1,873,432,000	1,330,831,000	1,249,850,000	1,841,539,000	1,522,893,000
2009	1,870,646,000	1,416,659,000	1,314,915,000	1,812,614,000	1,565,942,000
2010	1,810,620,000	1,252,063,000	1,294,065,000	1,911,522,000	1,573,156,000
2011	1,835,118,000	1,201,403,000	1,287,709,000	1,857,041,000	1,652,617,000
2012	1,719,600,000	1,271,997,000	1,200,327,000	1,849,598,000	1,646,865,000
2013	1,824,836,000	997,251,000	1,083,325,000	1,621,772,000	1,659,882,000
2014	1,670,080,000	897,762,000	973,360,000	1,626,955,000	1,603,700,000
2015	1,501,317,000	928,538,000	845,609,000	1,530,995,000	1,594,429,000

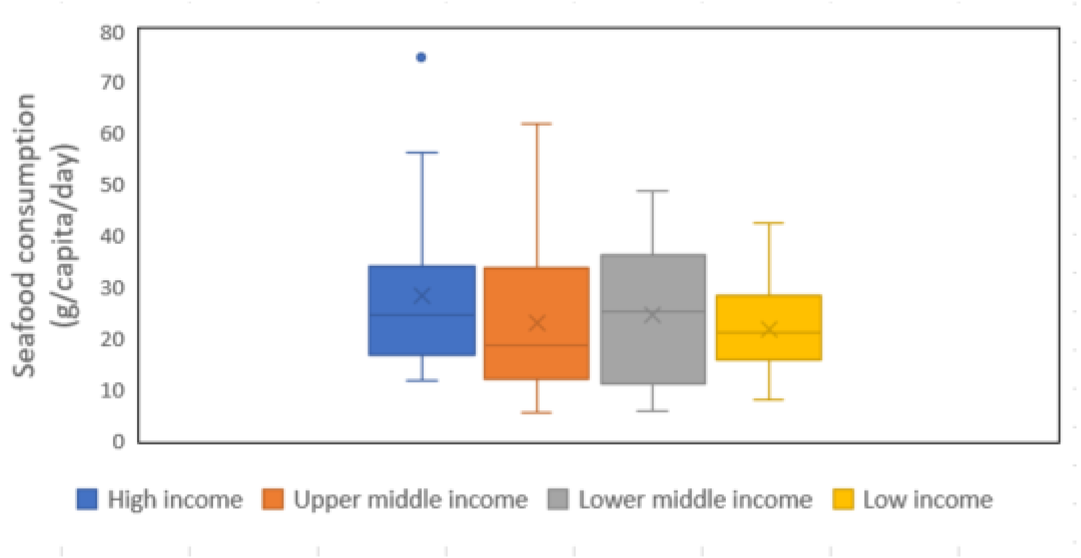

Figure S1. Per capita seafood consumption in 187 countries, which were taken from the Global Dietary Database (2019). This data could be broadly classified into four groups: (1) High income; (2) Upper middle income; (3) Lower middle income; and (4) Low income. Box plots display 10th, 25th, 50th, 75th and 90th percentiles, and individual data points outside the 10th and 90th percentiles. x represent the mean of per capita seafood consumption.
